# Landmark mediation survival analysis using longitudinal surrogate

**DOI:** 10.3389/fonc.2022.999324

**Published:** 2023-01-17

**Authors:** Jie Zhou, Xun Jiang, H. Amy Xia, Brian P. Hobbs, Peng Wei

**Affiliations:** ^1^ Department of Biostatistics and Pharmacometrics, Neuroscience Global Drug Development, Novartis, East Hanover, NJ, United States; ^2^ Center for Design and Analysis, Amgen, Thousand Oaks, CA, United States; ^3^ Department of Population Health, The University of Texas, Austin, TX, United States; ^4^ Department of Biostatistics, The University of Texas MD Anderson Cancer Center, Houston, TX, United States

**Keywords:** functional principal component analysis, landmark analysis, longitudinal analysis, mediation analysis, oncology, RECIST

## Abstract

Clinical cancer trials are designed to collect radiographic measurements of each patient’s baseline and residual tumor burden at regular intervals over the course of study. For solid tumors, the extent of reduction in tumor size following treatment is used as a measure of a drug’s antitumor activity. Statistical estimation of treatment efficacy routinely reduce the longitudinal assessment of tumor burden to a binary outcome describing the presence versus absence of an objective tumor response as defined by RECIST criteria. The objective response rate (ORR) is the predominate method for evaluating an experimental therapy in a single-arm trial. Additionally, ORR is routinely compared against a control therapy in phase III randomized controlled trials. The longitudinal assessments of tumor burden are seldom integrated into a formal statistical model, nor integrated into mediation analysis to characterize the relationships among treatment, residual tumor burden, and survival. This article presents a frameworkfor landmark mediation survival analyses devised to incorporate longitudinal assessment of tumor burden. *R* ^2^ effect-size measures are developed to quantify the survival treatment mediation effects using longitudinal predictors. Analyses are demonstrated with applications to two colorectal cancer trials. Survival prediction is compared in the presence versus absence of longitudinal analysis. Simulation studies elucidate settings wherein patterns of tumor burden dynamics require longitudinal analysis.

## 1 Introduction

Defined as the time from randomization until death from any cause, overall survival (OS) is considered the most reliable cancer endpoint for randomized controlled trials. Measurement of OS is unbiased and precise. Prolonging OS is considered the most convincing demonstration that an emerging cancer therapeutic intervention is superior to an existing standard of care. Despite these advantages, a long duration is required to sufficiently follow trial participants to observe OS. Especially in earlier stages, for which recent advances in surgical, radiation, and non-cytotoxic treatments have extended patient survival to an extent that there are now considerable numbers of patients alive 5 years after diagnosis (such as breast cancer and colon cancer). Authors report that the median duration of an industry sponsored phase III oncology trial that completes enrollment is approximately 48 months (1).

This latency period required to acquire sufficiently mature OS data effectively delays the confirmation of OS benefit. For many cancer types, radiographic tumor assessments are used to directly measure components of the disease and trigger treatment decisions in clinical practice. While a variety of tumor response criteria are considered appropriate for regulatory reviews, the revised Response Evaluation Criteria in Solid Tumors (RECIST) (2) is the predominate technique for quantifying changes in tumor burden following treatment for solid tumors. The RECIST criteria is a composite endpoint which characterizes changes in tumor size by the sum of longest diameters (LDSUM). Thought to be a surrogate endpoint for OS, changes in tumor burden are described by four levels of response for target and non-target lesions. For example, for target lesions, takingas reference the smallest LDSUM on study, observing a 20% increase yields the worst result of progressive disease (PD). The best result occurs with the disappearance of all target lesions, or complete response (CR). A 30% decrease from the baseline LDSUM yields a partial response (PR). A patient is considered to have stable disease (SD) if criteria for PD, PR, and CR are not satisfied.

Tumor assessments occur longitudinally over the course of study, typically planned to be acquired at 4-week or 8-week intervals until tumor progression. Planned trial statistical analyses, however, reduce the longitudinal curves to a single response level. At the patient level, the objective response indicates whether a patient experienced PR or CR during the course of study. At the cohort level, the objective response rate (ORR), the proportion of patients that achieved an objective response, is a commonly used primary endpoint for single-arm trials. Objective response is used alongside endpoints describing the duration of response to support applications for accelerated approval to the U.S. Food and Drug Administration.

In oncology settings, complex relationships exist among therapies, tumor response (often used as the basis for evaluation in phase II trials), and survival. Reducing the longitudinal tumor assessment to a single value, however, may not describe the cumulative effect of the patient’s tumor burden. Mediation modeling provides a framework for elucidating the mechanisms by which an intervention impacts an endpoint through a third intermediate response variable. The extent to which tumor response representsa reliable surrogate of survival can be measured through the application of mediation models, which decompose the total effect of an intervention into direct effect and indirect effects. In the context of oncologic drug development, the indirect effect defines the extent of survival benefit that is achieved from improving the objective response rate, while direct treatment effects characterize the extent of survival benefit attributable to all other factors. Authors have developed mediation models integrating categorical surrogate endpoints with OS (3, 4). However, to our knowledge a mediation model for OS that leverages the entire tumor assessment trajectory for each patient has not been developed.

Longitudinal data is often analyzed using the linear mixed effects models or generalized estimation equations (5, 6). Functional principal component analysis (FPCA) is another popular method that provides a powerful approach for modelling noisy and irregularly measured longitudinal data. Summary statistics derived from FPC scores yield dimension reduction of the trajectories, while preserving most of the information. Survival models with longitudinal predictors have been developed under the joint modeling framework (7, 8). Specifically, a mixed effects model with normal random effects is commonly assumed for the longitudinal observations. The hazard function or the survival probability is assumed to depend on the true longitudinal outcome at each time point. Naive two-stage approaches (9, 10) were first proposed for estimating the association between the longitudinal and survival outcomes. More advanced estimating procedures based on the Expectation-Maximization (EM) algorithm (11) and Markov chain Monte Carlo (MCMC) (12) were subsequently proposed to reduce bias in parameter estimation. Dynamic predictions of survival probabilities conditional on the available longitudinal data have been developed for joint models (13–15). The application of such joint models is challenging in practice, however. The fitting algorithms are computationally expensive and many underlying parametric distributional assumptions cannot be verified from the data. Furthermore, the extensions to estimate the time-varying effects of different types of longitudinal response variables are not straightforward.

Landmark analysis first proposed in Anderson et al. (16) provides a straightforward approach to approximate the association between longitudinal and OS outcomes at a sequence of landmark times. Landmark models have been developed to estimate the varying effect of a longitudinal predictor on the survival outcome and predict the survival outcomes dynamically (17). Cao et al. (18) calculated the cumulative effect of longitudinal outcome based on FPCs and applied the measure in models with binary disease outcomes. Yan et al. (19) used landmark analyses with FPCs derived based on moving time windows in predicting survival probabilities dynamically. Shi et al. (20) applied the FPCA based landmark analysis in estimating the effect of longitudinal measures of total cholesterol with respect to risk of coronary heart disease. Though it is not a comprehensive probability model of the longitudinaland the event time processes, landmark analysis circumvents the assumptions and computational burden associated with a joint model.

This article presents a method for quantifying the treatment mediation effects of tumor response on survival using longitudinally observed tumor measurements. *R* ^2^ effect-size measures (21, 22) are extended to the settingof longitudinal survival mediation. Different from the traditional product-based and difference-based mediation effect measures, which are based on coefficients estimated in regression models (23), R-squared type measures are derived from the extent of explained variation of survival outcomes by treatment and mediators. Several *R* ^2^ measures have been proposed in the literature. Kent and O’Quigley (24) derived an explained risk measure called “explained randomness” using Weibull models with entropy loss function. Korn and Simon (25)Korn and Simon (, 26) discussed explained risk measures for residual variation in survival analysis. To account for censoring in the survival data, authors have incorporated inverse probability censoring weights (27, 28), but the measures depend on the maximum follow-up time and could be sensitive to the values in the right tail of the survival distribution. To avoid any modification or approximation to the metric, Heller (29) proposed a measure called explained relative risk for the proportional hazards model, which is unaffected by non-informative censoring times. Shi et al. (30) compared the different *R* ^2^ measures for survival outcomes and suggested two *R* ^2^ measures proposed in Kent and O’Quigley (24) and Heller (29) that satisfy the properties conveyed by Royston (31). These methods have not be used to compare tumor burden (TB) trajectories with landmark analyses. We compare mediation analysis for OS using longitudinal measures of tumor response versus conventional approaches using the single-valued objective response. Measures are estimated using patient-level data acquired from two colorectal cancer studies [Goldberg et al. (32); Peeters et al. (33)], which we analyzed before as secondary analyses in different contexts [Hobbs et al. (34); 120 Zhou et al. (3); Zhou et al. (4)].

The remainder of this article is organized as follows. In Section 2.1, we introduce the varying coefficient models for the longitudinal outcomes and different response summary statistics. The landmark analysis models are discussed in Section refsec:osmod for estimating the time-varying effect of longitudinal outcomes and predicting survival probability for new patients. The *R* ^2^ measures are introduced in Section 2.3 for measuring the mediation effects of longitudinal tumor measurements. The models comparing different measures are applied to two colorectal cancer studies in Section 2.4. Comprehensive simulation studies are performed in Section 3 to evaluate the predictive performance of summary statistics of longitudinal measures under settings assuming various relationships among treatment, longitudinal and survival outcomes. Finally, we summarize the content and have some discussions in Section 5.

## 2 Methodology

Let *A*∈*A* indicate treatment arm. For simplicity, we present notation assuming only two treatment options *A*=(0,1) as extensions to three or more are relatively straightforward. *X* and *Z* denote covariate vectors for longitudinal and survival models, respectively. *X* and *Z* may represents different features or shared common variables. lationships among treatment, TB, and OS.

### 2.1 Longitudinal tumor burden

Let *Y*_*ij*_=*Y*_*i*_(*t*_*ij*_) denote the observed longitudinal measures of TB for the *i* th patient at visit time *t*_*ij*_ , *i*=1,⋯,*N* ; *j*=1,⋯,*m*_*i*_ . We assume *Y*_*ij*_ follows a varying coefficient model:


$$Y_{i}\left(t\right)=\eta_{0}\left(t\right)+D_{i}\left(t\right)+\epsilon_{i}\left(t\right)$$

(1) $$=\eta_{0}\left(t\right)+\eta_{1}\left(t\right)\times A_{i}+\eta_{2}\left(t\right)\times X_{i}+\epsilon_{i}\left(t\right),$$

Where *η*_0_(*t*) is the overall population mean trajectory of TB and *η*_1_(*t*) and *η*_2_(*t*) are the time-varying treatment and covariate effects on the tumor. The error term *ϵ*_*ij*_=*ϵ*_*i*_(*t*_*ij*_) is assumed to follow a Normal distribution *N*(0,*σ* ^2^) . Note that *D*_*i*_(*t*) is the true longitudinal trajectory with the overall mean trajectory removed, and therefore contains the patient level variability of TB.

### 2.2 Functional principal component analysis

Yao et al. (35) proposed an approach called Principal Components Analysis through Conditional Expectation (PACE) for sparse and irregularly measured longitudinal data. According to the Karhunen–Loeve decomposition, the patient level longitudinal trajectory *D*_*i*_(*t*) in Equation (1) can be decomposed as $D_{i}(t)=\sum_{k=1}^{\infty }\gamma_{ik}p_{k}(t)$ where *ρ*_*k*_(*t*) is the *k* th eigenfunction satisfying the orthonomal conditions: ∫^​^ *ρ*_*k*_(*s*)*ρ*_*l*_(*s*)*ds*=0 and ∫^​^ *ρ*_*k*_(*s*)^2^ *ds*=1 for any *k*≠*l* and *k*=1,⋯,*∞* . Parameter *γ*_*ik*_=∫^​^(*Y*_*i*_(*t*)−*η*_0_(*t*))*ρ*_*k*_(*t*)*dt* is the functional principal component (FPC) score corresponding to the *k* th eigenfunction.

The variability contained by the functional components decreases as *k* increases. Usually the trajectories can be well approximated by the first finite number (say *K* ) of components. Therefore we estimate the smoothed trajectories


$${\hat{D}}_{i}\left(t\right)=\sum_{k=1}^{K}{\hat{\gamma }}_{ik}\rho_{k}\left(t\right),$$

where ${\hat{\gamma }}_{ik}$ is the FPC score estimated through conditional expectation. The estimation of the overall mean function, eigenfunctions, and FPC scores can be achieved in the R package “fdapace”.

To make full use of the longitudinal TB information, we consider two approaches to estimation. One approach uses the first *K* FPC scores that capture at least 90% of the total outcome variability. The FPC scores summarize a patient trajectory’s proximity to the patterns described by the eigenfunctions. Restricting estimation to 90% of variability explained captures most information of the longitudinal outcome. The other approach summarizes the cumulative effect of longitudinal outcomes. Let *τ*_*i*_ denote the upper limit of observational time of the longitudinal measures for patient *i*. We define the integrated smoothed outcome of *D*_*i*_(*t*) as ${\hat{ID}}_{i}=\int_{0}^{\tau_{i}}{\hat{D}}_{i}\left(s\right)ds$ , which can be calculated through numerical integration.

### 2.3 Time-varying models for overall survival

Let *T* denote overall survival duration since trial enrollment. To evaluate treatment effect on OS after adjusting for TB, we assume *T* follows a proportional hazards (PH) model (36)


$$h(t|A_{i},R_{i},Z_{i})=h_{0}\left(t\right)\times \exp\;\{\beta_{1}A_{i}+\beta_{2}Z_{i}+\alpha R_{i}\},$$

where *h*_0_(*t*) is the baseline hazard function. The baseline hazard is positive and can be estimated nonparametrically. *R*_*i*_ denotes the tumor response surrogate measure. For conventional mediation analysis, *R*_*i*_ may be the binary indicator of an objective RECIST tumor response. Leveraging the longitudinal model, one could use the first *K* FPC scores as *R*_*i*_ or the fully integrated smoothed outcome ${\hat{ID}}_{i}$ ) of TB discussed in Section 4.2. Of note, the classical causal mediation analysis model for survival outcomes assumes no unmeasured confounders for the exposure-outcome (A-T), exposure-mediator (A-R), and mediator-outcome (R-T) relationships VanderWeele (23). As our goal here is prediction rather causal inference, we do not make such assumptions. Nevertheless, adjusting covariates, i.e., potential confounders, in the PH models is expected to improve the prediction accuracy Vandenberghe et al. (37).

The true survival time *T* is subject to right censoring. Let *C*_*i*_ denote the censoring duration of patient *i*. For modeling justification, we need to assume that the censoring mechanism is random or non-informative. Therefore, *T*_*i*_ and *C*_*i*_ are independent conditional on the treatment *A*_*i*_ , covariates *Z*_*i*_, and longitudinal outcome variable *R*_*i*_ . The observed survival outcome for patient *i* includes an observed survival duration ${\tilde{T}}_{i}=min\left(T_{i},C_{i}\right)$ and censoring indicator *δ*_*i*_=*I*(*T*_*i*_ *łeqC*_*i*_) .

With complete dataset $O=\left\{\left({\tilde{T}}_{i},\delta_{i},A_{i},X_{i},Z_{i},R_{i}\right),i=1,\cdots ,N\right\}$ , the overall effects of treatment and covariates can be estimated with the traditional nonparametric Cox PH model. However, this model assumes that the effects of treatment, TB, and covariate on OS are fixed over time. Ignoring the possibility of time-varying effects, this strong assumption is usually difficult to justify. easures. This limitation can be addressed by adopting a landmark analysis approach.

#### 2.3.1 Landmark analysis

Let 0<*L*_1_<*L*_2_<⋯<*L*_*P*_<*τ* denote a sequence of landmark times of interest, where *τ*=*max* (*τ*_1_,⋯,*τ*_*N*_) is an upper limit of the longitudinal follow-up time of all patients. For each landmark time *L*_*p*_ , only patients in the risk set at *L*_*p*_ are used for estimation. Define $t_{i}^{\left(p\right)}=\{t_{ij}:t_{ij}<L_{p},j=1,\cdots ,m_{i}\}$ to be the observational time points before landmark time $L_{p}.\;Y_{i}^{\left(p\right)}$ denotes the corresponding longitudinal outcomes with measurement time in $t_{i}^{\left(p\right)}$ . The response summary variable $R_{i}^{\left(p\right)}$ is then derived based on available data $Y_{i}^{\left(p\right)}$ at landmark time *L*_*p*_ . Specifically, a conventional model using binary objective response will allow different patterns for responders after the response is observed, while non-responders will assume constant values at 0 for all time. When is defined based on FPCA, as discussed in Section 4.2, FPCA can be applied according to a moving time window (19).

Initially, FPCA is performed with all available longitudinal data using the maximum time window (0,*τ*) . The estimated overall mean function ${\hat{\eta }}_{0}(t)$ , as well as the eigenvalues and eigenvectors corresponding to the first K FPCs are saved. Then at each landmark time *L*_*p*_ , FPC scores are recalculated as ${\hat{\gamma }}_{ík}^{\left(p\right)}$ based on available longitudinal outcomes $Y_{i}^{\left(p\right)}$ . The integrated score at landmark tim*L*_*p*_ is calculated as *R*_*i*_ $\hat{ID_{i}^{\left(p\right)}}=\int_{0}^{\tau_{i}^{p}}{\hat{D}}_{i}\left(s\right)ds$ ,where $\tau_{i}^{p}=\min\;(\tau_{i},L_{p})$ .

The landmark data at *L*_*p*_ is defined as $O^{\left(p\right)}=\{\left({\tilde{T}}_{i},\delta_{i},A_{i},X_{i},Z_{i},R_{i}^{\left(p\right)}\right):{\tilde{T}}_{i}>L_{p};i=1,\cdots ,N\}$ and the survival model for *T*−*L*_*P*_ given *O* ^(*p*)^ is defined as:


(2) $$h(t|O^{\left(p\right)})=h_{0}^{\left(p\right)}\left(t\right)\times \exp\;\{\beta_{1}\left(L_{p}\right)\cdot A_{i}+\beta_{2}\left(L_{p}\right)\cdot Z_{i}+\alpha \left(L_{p}\right)\cdot R_{i}^{\left(p\right)}\},$$

where $h_{0}^{\left(p\right)}\left(t\right)$ is the nonparametric baseline hazard function evaluated at time *L*_*p*_+*t* . Trajectories of estimated coefficients ${\hat{\beta }}_{1}\left(\cdot \right)$ , ${\hat{\beta }}_{2}\left(\cdot \right)$ and $\hat{\alpha }\left(\cdot \right)$ represent the changing patterns of the effects of treatment, covariate, and TB measures on overall survival. These model features provide estimates for time-varying coefficients at landmark time points of interest. Moreover, the estimated models can be used for prediction at each landmark time, which will be discussed in the next section.

#### 2.3.2 Prediction for new patients

After estimating the model in (2) for all the landmark times, we can dynamically predict survival probabilities for new patients at each landmark time. Specifically, the conditional survival probability for a new patient *N*+1 , who survivedlonger than landmark time *L*_*p*_ and had data $O_{N+1}^{\left(p\right)}=\left(A_{N+1},Z_{N+1},R_{N+1}^{\left(p\right)}\right)$ , can be written as


$$\hat{S}(L_{p}+t|L_{p},O_{N+1}^{\left(p\right)})=Pr\{T_{N+1}>L_{p}+t|T_{N+1}\rangle L_{p},A_{N+1},Z_{N+1},R_{N+1}^{\left(p\right)}\}$$

,(3) $$={\hat{S}}_{0}^{\left(p\right)}{\left(t\right)}^{\exp\;\{{\hat{\beta }}_{1}\left(L_{p}\right)\cdot A_{N+1}+{\hat{\beta }}_{2}\left(L_{p}\right)\cdot Z_{N+1}+\hat{\alpha }\left(L_{p}\right)\cdot R_{N+1}^{\left(p\right)}\}}$$

where ${\hat{S}}_{0}^{\left(p\right)}\left(t\right)=\exp\;\{-\int_{0}^{t}h_{0}^{\left(p\right)}\left(s\right)ds\}$ is the baseline survival function corresponding to landmark time *L*_*p*_ .

The predictive power of various tumor burden summaries, *R*_*i*_, discussed in Section 4.1 can be evaluated by comparing the resulting area under curves (AUC):


(4) $$AUC_{p}\left(u\right)=Pr\{S(L_{p}+t|L_{p},O_{i}^{\left(p\right)})<S(L_{p}+t|L_{p},O_{j}^{\left(p\right)})|L_{p}\langle T_{i}\leq L_{p}+u,T_{j}\rangle L_{p}+u\}.$$

The estimated value ${\hat{AUC}}_{p}\left(u\right)$ can be obtained by plugging the predicted survival probabilities (3) in equation (4). Models with larger values of ${\hat{AUC}}_{p}\left(u\right)$ facilitate more accurate predictions at landmark time *L*_*p*_ .

Brier score (38) is another measure of prediction performance characterized by the mean squared error for the predicted survival probabilities calculated in equation (3). This measure is useful for model comparison in simulation studies where the true survival probabilities are known Shi et al. (20). However, the Brier score cannot be directly applied in real data analysis with right-censored observations unless modified using the inverse probability of censoring weighting method Graf et al. (27), which can be numerically unstable Seaman and White (39).

### 2.4 R2 measures for mediation effect

*R* ^2^ effect-size measures were originally proposed to assess the variance accounted for in mediation models with uncensored continuous outcomes (21). According to Yang et al. (22) and Shi et al. (30), three differentregression models need to be fit to calculate the *R* ^2^ measures for mediation effects: (1) a model with the independent variable of interest, (2) a model with the mediators and (3) a full model with all predictors. Specifically, we fit the following three PH models:


(5) $$h(t|A_{i})=h_{0}^{D}\left(t\right)\times \exp\;\{\beta_{1}^{D}A_{i}\},$$

(6) $$h(t|R_{i})=h_{0}^{M}\left(t\right)\times \exp\;\{\alpha^{M}R_{i}\},$$

(7) $$h(t|A_{i},R_{i})=h_{0}^{F}\left(t\right)\times \exp\;\{\beta_{1}^{F}A_{i}+\alpha^{F}R_{i}\},$$

where the superscripts ‘D’, ‘M’ and ‘F’ denote the “direct”, “mediated” and “full” models. These three models are used to distinguish the baseline hazard and regression coefficients. Let $R_{T,D}^{2}$ denote the total variation of survival time *T* explained by treatment. This can be derived based on model (5). Similarly, let $R_{T,M}^{2}$ calculated based on model (6) represent the variation of *T* explained by the response summary variable, and $R_{T,F}^{2}$ calculated based on the full model (7) is the the variation of *T* explained by the treatment and response summary variables conjointly. The R-squared measure of mediated effect is then calculated as


(8) $$R_{med}^{2}=R_{T,D}^{2}+R_{T,M}^{2}-R_{T,F}^{2}.$$

The proportion of mediated effect with respect to the total treatment effect on survival is defined as the shared over simple (SOS) effect. SOS effect is calculated as $SOS=R_{med}^{2}/R_{T,D}^{2}$ . Provided a nonzero total effect and non-negative direct and mediated effects (i.e. $0\leq R_{med}^{2}\leq R_{T,D}^{2}$ and $R_{T,D}^{2}>0$ ), the mediation effect of the response summary variable increases as *SOS* increases from 0 to 1.

To calculate the *R* ^2^ measures in Equation (8), Shi et al. (30) compared five available approaches and suggested the $R_{b}^{2}$ proposed by Heller (29) as well as $R_{w}^{2}$ proposed by [15] which is based on the five properties suggested in (31) for evaluating the *R* ^2^ measures in survival models. n the manuscript, and results with other approaches can be found in the **Supplementary Materials**. Let *θ* denote the vector of regression coefficients in models (5)-(7) and *P*_*i*_ denote the corresponding vector of predictor values for patient *i* , *i*=1,⋯,*N* . The R-squared measure $R_{b}^{2}$ derived by Heller (29), which they referred to as the explained relative risk, is calculated as


$$R_{b}^{2}=\frac{log\left[N^{-1}\sum_{i}exp\;({\hat{\theta }}^{'}\tilde{P_{i}}\right)]}{1.5772+log\left[N^{-1}\sum_{i}exp\;({\hat{\theta }}^{'}\tilde{P_{i}}\right)]-1},$$

where $\tilde{P_{i}}$ is the covariate value centered around zero and $\hat{\theta }$ is the maximum partial likelihood estimate for *θ* . Note that 1.5772 is the approximate value for the entropy for the covariate model under the extreme value distribution. The other measure $R_{w}^{2}$ using the approach proposed by [15] was developed from a Weibull model as an approximation to the explained risk based on the product moment correlation coefficient with a standard normal error variance:


$$R_{w}^{2}=\frac{{\hat{\theta }}^{'}\hat{\Sigma_{P}}\hat{\theta }}{1+{\hat{\theta }}^{'}\hat{\Sigma_{P}}\hat{\theta }},$$

where $\hat{{\text{Σ}}_{P}}$ is the estimated variance covariance matrix of the covariate vector.

Note that the R-squared based approaches require large sample sizes in practice. Based on our numerical studies, the estimated *SOS* effects using the above approaches are not always restricted to the interval [0,1], especially when the total treatment effects are small while the sample sizes are moderate.

## 3 Colorectal cancer studies

This section applies the landmark survival mediation analysis to two colorectal cancer studies. The predictive power of various tumor burden measures are compared at different landmark times. Landmark times were selected as the 10th, 20th,…,90th quantiles of event times observed in each study. Estimated time-varying coefficients of treatment and baseline LDSUM were compared among four models: (i) model with no response variable adjusted; (ii) model adjusting binary response; (iii) model adjusting the first three FPC components that explained more than 99% variability in the outcome and (iv) model adjusting the integrated smoothed TB trajectory.

### 3.1 Goldberg study

The colorectal cancer study reported by Goldberg et al. (32) included 795 patients with metastatic colorectal cancer who had not been treated previously for advanced disease. Secondary analysis of the trial data has been reported Hobbs et al. (34). These patients were enrolled in the study between May 1999 and April 2001 and had a median follow-up of 20.4 months (88 weeks). There were three treatment arms including irinotecan and bolus fluorouracil plus leucovorin (IFL), oxaliplatin and infused fluorouracil plus leucovorin (FOLFOX), or irinotecan and oxaliplatin (IROX). FOLFOX and IROX were two new regimens under investigation while IFL was considered as the standard of care.

**Figure 1:**
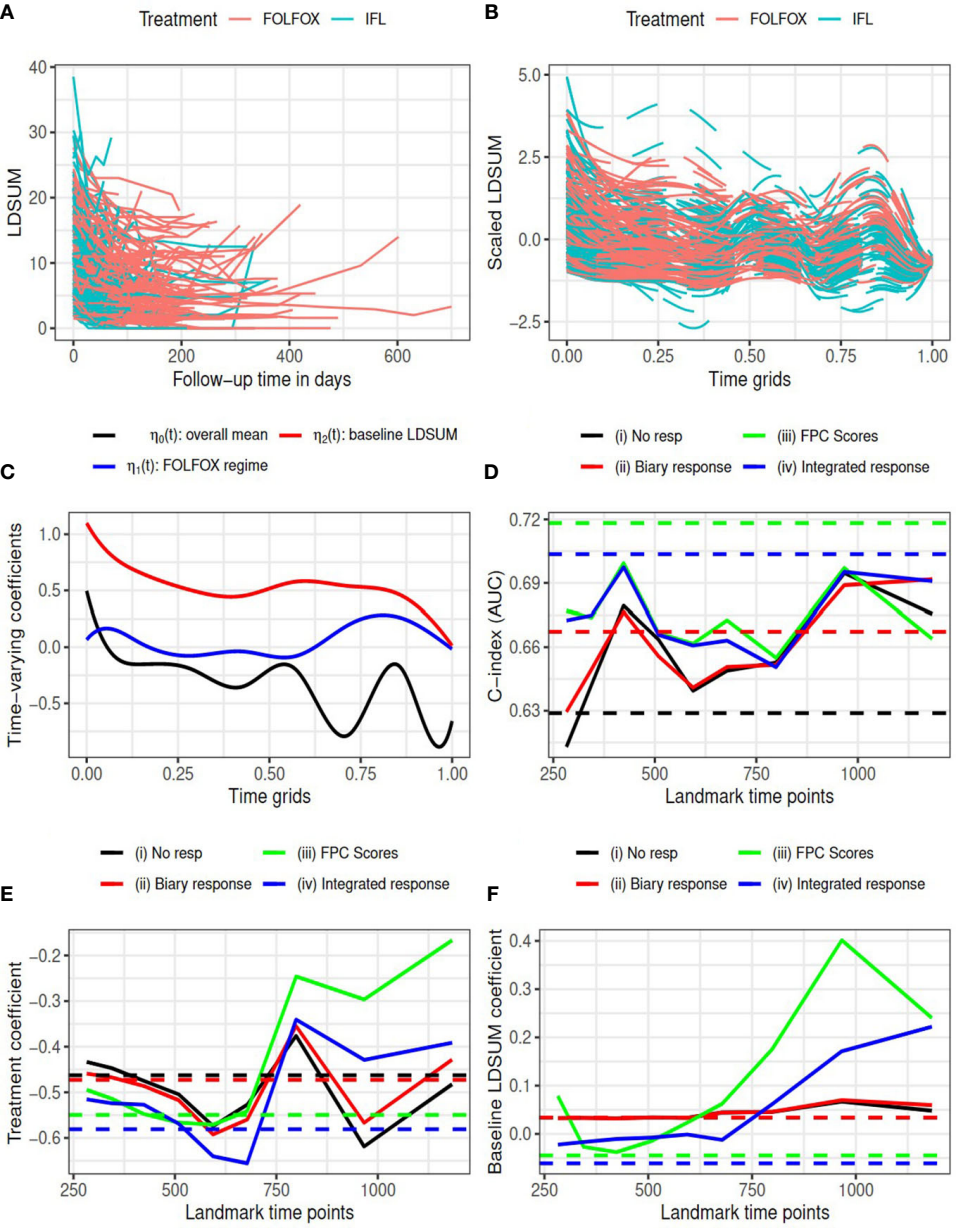
Goldberg study: **(A)** observed LDSUM by treatment groups; **(B)** smoothed (solid lines) and predicted (dashed lines) values for scaled LDSUM based on FPCA; **(C)** estimated varying coefficients in functional data regression analysis; **(D)** AUC values with different response measures; (e/f) estimated coefficient for treatment/baseline LDSUM in survival model. Solid lines are based on analysis and dashed lines are based on complete data in **(D–F)**.

Our case study compares the tumor response and overall survival outcomes between patients who received the FOLFOX regime (treatment group) and the standard of care IFL (control group). Analysis was limited to patients with at least three measurements of tumor burden as defined by RECIST LDSUM. As a result, the analysis set included a total of 311 patients (157 in treatment group and 154 in control group). Tumor burden was evaluated at each treatment cycle (every 2 weeks). (This is the description in the study, but we assumed 2 wks for each cycle in data. Do we need to note here)? Longitudinal LDSUM measures are plotted by treatment groups in **Figure 1A**. The median number of follow-up visits is five. Baseline LDSUM is included as a covariate in models for both longitudinal LDSUM and OS. The baseline LDSUM ranged from 1.5 to 38.5 centimeters (cm) with mean of 10.18 cm in the treatment group compared with 10.11 cm in the control group.

According to RECIST criteria, patients in the treatment group experienced more reductions in tumor burden than patients in the control group: 114 (72.6%) responders (14 CR and 100 PR) among patients receiving FOLFOX versus 93 (60.4%) responders (5 CR and 88 PR) for patients receiving IFL. We performed FPCA on the scaled longitudinal LDSUM. The time scale was map into [0, 1] by dividing the observational time by the maximum follow-up duration observed. **Figure 1B**, plots the smoothed outcome up to the last observational time. Solid lines depict observed domain for each patient, while dashed lines are used to depict the predicted trajectories for each patient. Functional data regression was fit using equation (1) to the smoothed trajectories yielding the estimated intercept and coefficients for treatment and baseline LDSUM in plotted in **Figure 1C**. Based the resultant estimated coefficient trajectories, we find that the treatment effect on the longitudinalLDSUM measures fluctuates around zero after adjusting for baseline tumor burden. Patients with larger baseline LDSUM also had larger LDSUM measures but the correlation decreases with time.

The C-index (or AUC) evaluated at median survival time is computed and compared for the four models with complete data (dashed lines) at each landmark time (solid lines) in **Figure 1D**. With complete data, models (iii) and (iv) based on the FPCs resulted in larger AUC values than model (ii) which described changes in TB as the binary objective response. All the three models containing tumor response information yielded larger AUC than model (i). Models (iii) and (iv) yielded larger AUC values than models (i) and (ii) for landmark time points before 800 days, while the AUC values are comparable beyond 800 days. **Figures 1E, F** present the estimated coefficients for treatment and baseline LDSUM for four models at eachlandmark time. The point estimates based on the complete dataset are marked as dashed lines in the plot. Patients receiving the FOLFOX regime experienced lower hazard of death compared to patients receiving IFL after adjusting baseline LDSUM measures andresponse based on all four models.

**Table 1** reported the estimated *R* ^2^ measures for models (ii)-(iv). Note the estimated measures fall below zero occasionally in this data due to the small sample size and mode *R* ^2^ rate total effects. All *R* ^2^ measures are close to zero, indicating limited mediated effect of the response summary variables. With limited differentiation between treatment and the longitudinal LDSUM, as estimated in **Figure 1C**, the mediation path from treatment to response to OS is not identified from the data.

**Table 1:** Colorectal studies: estimated *R* ^2^ and SOS measures for mediation effects.

		(ii) Binary response		(iii) FPC scores		(iv) Integrated response	
Study	measure	$R_{med}^{2}$	SOS	$R_{med}^{2}$	SOS	$R_{med}^{2}$	SOS
Goldberg	$R_{b}^{2}$	-0.00992	-0.22303	-0.00106	-0.02394	-0.00684	-0.15374
	$R_{w}^{2}$	-0.00993	-0.19275	0.00457	0.08869	-0.00613	-0.11905
Amgen	$R_{b}^{2}$	-0.00001	-0.00447	0.00114	0.68428	0.0009	0.54028
	$R_{w}^{2}$	0.0002	0.10568	0.00069	0.35978	0.00118	0.61047

SOS: the shared over simple effect. It is defined as the ratio of R-squared measures of the mediated and the total treatment effects on OS.

### 3.2 Study of Panitumumab

**Figure 2:**
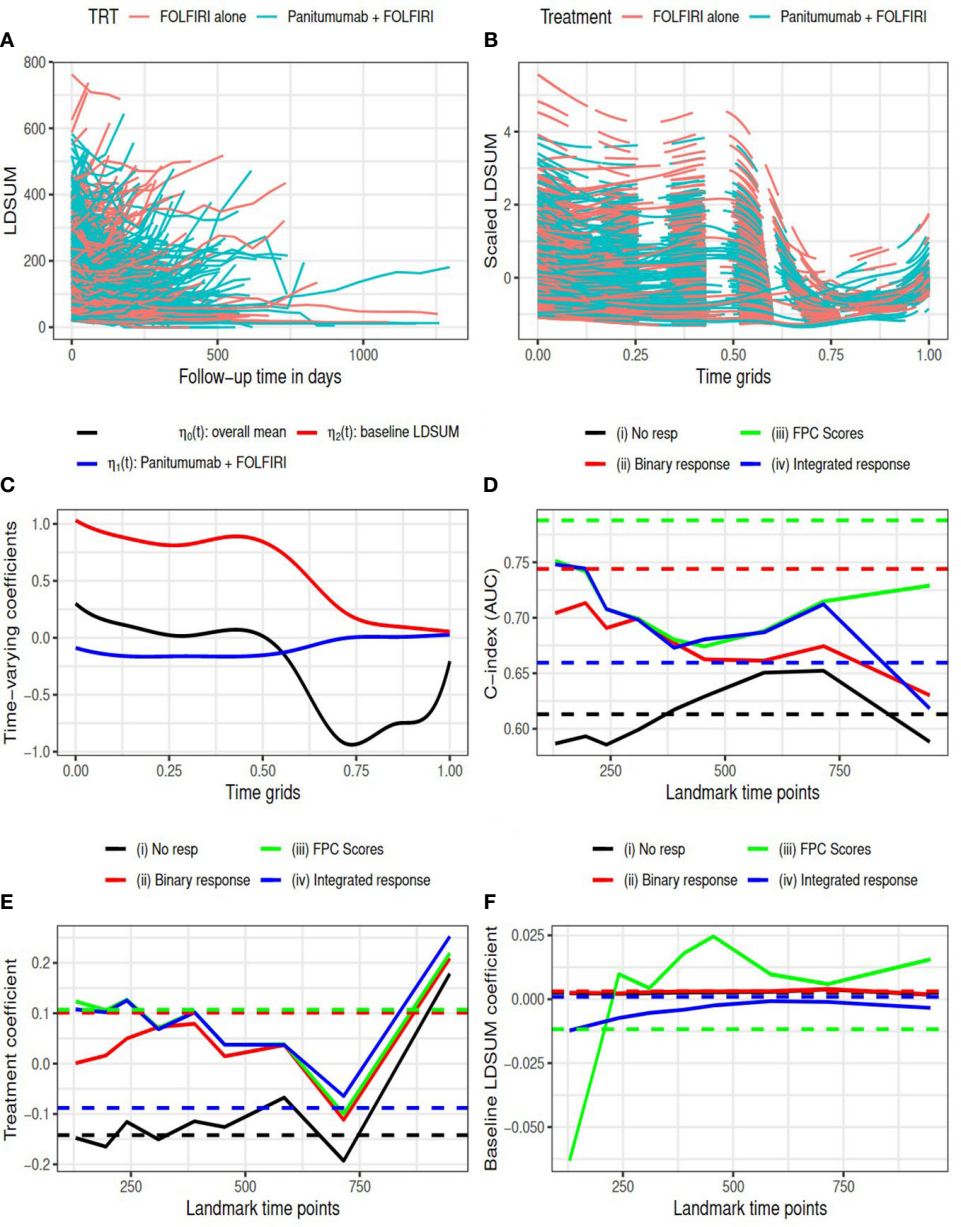
Amgen colorectal cancer study: **(A)** observed LDSUM by treatment groups; **(B)** smoothed (solid lines) and predicted (dashed lines) values for scaled LDSUM based on FPCA; **(C)** estimated varying coefficients in functional data regression analysis; **(D)** AUC values with different response measures; (e/f) estimated coefficient for treatment/baseline LDSUM in survival model. Solid lines are based on analysis and dashed lines are based on complete data in **(D–F)**.

The other colorectal cancer study was sponsored by Amgen (33). The study enrolled 1186 patients with Metastatic Colorectal Cancer (mCRC). Patients were randomized to receive either standard treatments of FOLFIRI (control group) or FOLFIRI plus Panitumumab (treatment group). The median follow-up time of the patients was 59 weeks. Our case study uses data made available on Project Data Sphere (PDS) (40), which comprised approximately 80% of patient-level data reported in the completed study. Analyses included patients with at least 2 measures of LDSUM during follow-up, yielding 841 patients (417 in control group and 424 in treatment group) for analysis. All patients with measurable disease at the baseline central review had their objective tumor response assessed every 8 weeks until progressive disease or death. The longitudinal LDSUM measures are plotted in **Figure 2A**. The median number of follow-up visits is four. Similar to analysis of the Goldberg study, we include the baseline LDSUM measures as predictors in models for both longitudinal LDSUM and OS. The baseline LDSUM ranged from 20 to 762 millimeter (mm) with an average size of target tumor at baseline of 168.9 mm and 164.1 mm in the treatment and control groups, respectively.

The resultant RECIST objective response rate in the treatment group is around 30%, which is much higher than the response rate in control group (12.5%). **Figure 2B** presents the predicted smoothed trajectories of longitudinal LDSUM. Estimates of the time-varying intercept and coefficient effects for treatment and baseline LDSUM are shown in **Figure 2C**. The plots demonstrate that the addition of Panitumumab had the effect of decreasing the LDSUM early following treatment. The effect, however, diminished with further follow-up. Similarly, patients with larger baseline LDSUM maintained larger LDSUM measures early on, The baseline effect of LDSUM also decreased to zero with time.

C-index (or AUC) evaluated at median survival time is compared among four models with complete data (dashed lines) and at each landmark time (solid lines) in **Figure 2D**. With complete data, model (iii) had the largest AUC and model (ii) with best response had larger AUC than model (iv). Models (ii), (iii) and (iv), which contained response information, were found to have larger AUC than model (i) at all the landmark time points.

In **Figures 2E, F**, the estimated coefficients for treatment and baseline LDSUM based on the four models were compared at each landmark time. The point estimates based on the complete dataset are marked as dashed lines in the plot. The treatment coefficients was negative in model (i) and positive in the other three models adjusted for response variables. The difference here indicated that the protective effect of additional Panitumumab on survival outcome strongly depended on the response information. The coefficients for baseline LDSUM were close to zero and the estimated values from model (iii) had larger variability.

The *R* ^2^ measures are listed in **Table 1**. The estimated *SOS* effects are close to zero for model (ii) and between 0.35 and 0.7 for models (iii) and (iv). This suggests that 35% to 70% of the treatment effect on OS is mediated by the longitudinal TB measures. Additionally, the FPC scores capture this mediation effect more efficiently than the conventional RECIST objective response. on effect during the follow-up between 300 to 700 days.

## 4 Simulation

### 4.1 Data generation

This section presents a simulation study devised to compare the four models (i)-(iv) discussed in Section 3. Using estimates from our case studies, we assume that the true trajectory of the longitudinal outcome was *η*_0_(*t*)+*η*_1_(*t*)*A*+*η*_2_(*t*)*X* with range for longitudinal follow-up time of *t*∈(0,1) Treatment *A* was generated from the Bernoulli distribution with probability of 0.5. Covariate matrix *X* was generated from the standard Normal distribution. For the time-varying coefficients, we used the estimated overall mean from the Goldberg study for *η*_0_(*t*) , and assume different time varying coefficients *η*_1_(*t*) and *η*_2_(*t*) for treatment and covariate *X* based on a cubic spline functions. Specifically, we defined four cubic B-spline basis function (*B*_1_(*t*),⋯,*B*_4_(*t*)) in the range of *t*∈(0,1) Model coefficient functions were determined by $\eta_{j}\left(t\right)=\sum_{l=1}^{4}\xi_{jl}B_{l}\left(t\right)$ .

We generate the true event time *T* from the survival model


$$h(t|A,Z,D\left(\tau \right))=h_{0}\left(t\right)\times \exp\;\{\beta_{1}A+\beta_{2}Z+\alpha \int_{0}^{\tau }D\left(s\right)ds\},$$

where *D*(*s*)=*η*_1_(*s*)*A*+*η*_2_(*s*)*X* is the true longitudinal trajectory after removing the overall mean function and *D*(*τ*) includes the history of *D*(*s*) up to time *τ*=*min* (*T*,1) . The baseline hazard function assumed Weibull distribution with shape 1.682 and scale 1.024, which were estimated from the Goldberg data. Covariate *Z* was generated from the standard Normal distribution.

Since the longitudinal predictor in the survival model is time-dependent when *T*<1 , we generated the event time using the following procedures. First, we set a sequence of grids 0=*s*_0_<*s*_1_<⋯<*s*_*M*_=1 , where *s*_*m*_−*s*_*m*−1_=0.001 . At each grid *s*_*m*_, theintegration part in the model can be approximated using the numerical integration. Specifically, we approximate $R\left(s_{m}\right)=\int_{0}^{s_{m}}D\left(s\right)ds$ by


$$R\left(s_{m}\right)\approx \tilde{R}\left(s_{m}\right)=\sum_{l=1}^{m}(D\left(s_{l}\right)-D\left(s_{l-1}\right))\left(s_{l}-s_{l-1}\right),$$

and the cumulative hazard function evaluated at time grid *s*_*m*_ can be approximated as


$$H\left(s_{m}\right)\approx \tilde{H}\left(s_{m}\right)=\exp\;(\beta_{1}A+\beta_{2}Z)\sum_{l=1}^{m}(s_{l}-s_{l-1})\left\{h_{0}\left(s_{l}\right)e^{\alpha \tilde{R}\left(s_{l}\right)}-h_{0}\left(s_{l-1}\right)e^{\alpha \tilde{R}\left(s_{l-1}\right)}\right\}.$$

The survival probability at time *s*_*m*_ is then $S\left(s_{m}\right)\approx \exp\;(-\tilde{H}\left(s_{m}\right))$ . For *T*≥1 , the cumulative hazard function is:


$$H\left(T\right)=\tilde{H}\left(s_{M}\right)+\exp\;\{\beta_{1}A+\beta_{2}Z+\alpha \tilde{R}\left(s_{M}\right)\}\int_{1}^{T}h_{0}\left(u\right)du.$$

To generate event time *T* that follows the desired distribution, we first generate *U*∼*Unif*(0,1) , and compare it with the survival probability *S*(*s*_*M*_) . If *U*>*S*(*s*_*M*_) , the event time *T*=*max* {*s*_*m*_:*S*(*s*_*m*_)≥*U*} , otherwise, *T* has closed form solution


$$T=H_{0}^{-1}\left\{H_{0}\left(s_{M}\right)-\frac{log\;(U)+\tilde{H}\left(s_{M}\right)}{exp\;(\beta_{1}A+\beta_{2}Z+\alpha \tilde{R}\left(s_{M}\right))}\right\},$$

Where $H_{0}\left(s\right)=\int_{0}^{s}h_{0}\left(u\right)du$ is the cumulative baseline hazard function and $H_{0}^{-1}$ is its inverse function.

We then generate independent censoring time *C* from uniform distribution *Unif*(0,10) , and the observed survival outcome as $\tilde{T}=\min\;(T,C)$ and *δ*=*I*(*T*≤*C*) . The longitudinal follow-up time *t*=(*t*_1_,⋯,*t*_*m*_) was randomly selected as the time grids on the interval $\left(0,\tilde{\tau }\right)$ , where $\tilde{\tau }=\min\;(1,\tilde{T})$ , and the number of total post-baseline visits *m* was generated from Poisson distribution with mean of 5. The observed longitudinal observations represent the true trajectory *η*_0_(*t*)+*D*(*t*) evaluated at visit time *t* plus error terms generated from *N*(0,*σ* ^2^=0.01) . Only patients with at least 3 post-baseline observations (*m*≥*q*3 ) were included in analysis.

### 4.2 Simulation settings and results

Our simulation study assumed five fundamentally different relationships among the treatment, longitudinal and survival outcomes. The coefficients for longitudinal and survival models are listed in **Table 2** for each setting. In the first two settings ‘a1’ and ‘a2’, there is only direct treatment effect on survival outcome with the response effect on OS assumed to be zero (i.e. *α*=0 ). The treatment and the longitudinal outcomes are not related in ‘a1’, while in setting ‘a2’, treatment has positive effect on the longitudinal outcome. Setting ‘b’ is another typical case where all the treatment effect on survival is mediated through the longitudinal response outcome. Setting ‘c’ has both direct and indirect treatment effects on the survival outcomes, while setting ‘d’ is a null case where treatment, longitudinal response and survival outcomes assume no dependence.

**Table 2:** Simulation settings and coefficients values.

Settings	Longitudinal model		Survival model		
	*ξ*_1_ (A)	*ξ*_2_ (X)	*β*_1_ (A)	*β*_2_ (Z)	*α* (R)
a1: direct effect only	(0,0,0,0)	(0.6,0.5,0.5,0.6)	-1	1	0
a2: direct effect only	(0,-0.2,-0.25,-0.7)	(0.6,0.5,0.5,0.6)	-1	1	0
b: indirect effect only	(0,-0.2,-0.25,-0.7)	(0.6,0.5,0.5,0.6)	0	1	10
c: both effects exist	(0,-0.2,-0.25,-0.7)	(0.6,0.5,0.5,0.6)	-1	1	10
d: no effect exists	(0,0,0,0)	(0.6,0.5,0.5,0.6)	0	1	0

**Figure 3:**
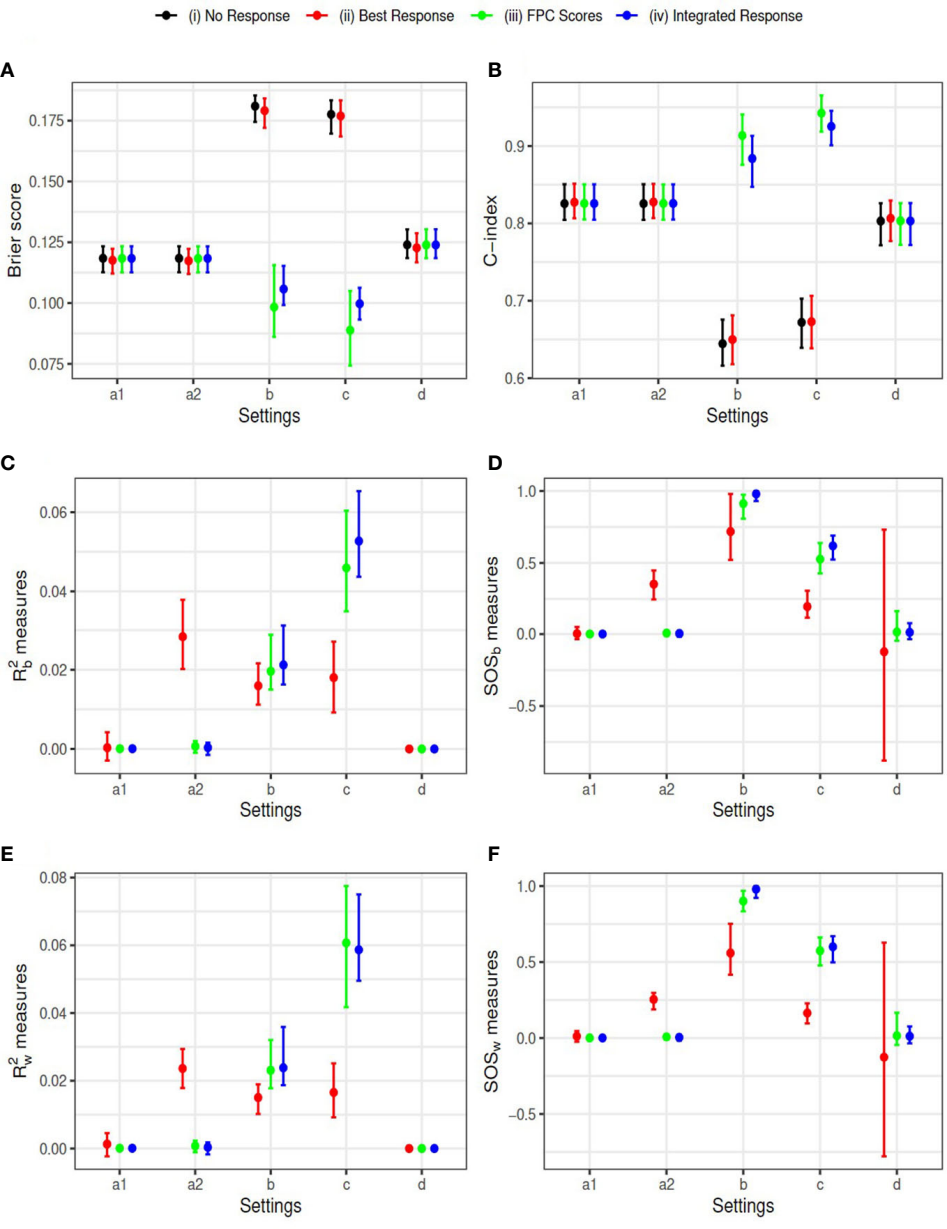
Simulation Results: Y-axis corresponds to **(A)** Brier score, **(B)** C-index, **(C)** R^2_b measure, **(D)** SOS_b measure, **(E)** R^2_w measure, and **(F)** SOS_w measure, respectively. Simulation settings include: direct effect only (settings a1 and a2), indirect effect only (setting b), both effects exist (setting c), and neither effect exists (setting d).

We used sample size *N*=1000 and 100 replications for each setting. The censoring time was generated from Uniform distribution *U*(0,10) , resulting in censoring proportions between 10% and 40%. Results comparing the four models based on the complete data are summarized in **Figure 3**. The mean and the 2.5% and 97.5% percentiles of the Brier scores and the C-index (AUC) evaluated at the median survival time are plotted in subfigures (a) and (b), respectively. The FPCA based models (iii) and (iv) have smaller Brier scores and larger AUCs in settings (b) and (c), where there is non-zero indirect treatment effect on survival through longitudinal outcome. The estimated *R* ^2^ measure and *SOS* effects using method $R_{b}^{2}$ are plotted in subfigures (c) and (d) with first and third quartiles. Subfigures (e) and (f) describe method $R_{w}^{2}$ . The results using the two approaches are similar. *SOS* estimated for models (iii) and (iv) are closer to 1 (the truth), in setting (b) than model (ii). In addition, larger variation of SOS is observed for model (ii) in some settings, especially in the setting (d), where treatment neither impacts the longitudinal LDSUM nor OS. Summary statistics of the estimated coefficients *β*_1_ and *β*_2_ can be found in the **Supplementary Materials**. The mean estimates of the four models are close to the truth in settings ‘a1’, ‘a2’ and ‘d’, while models (iii) and (iv) have smaller bias than model (ii) in settings (b) and (c).

**Figure 4:**
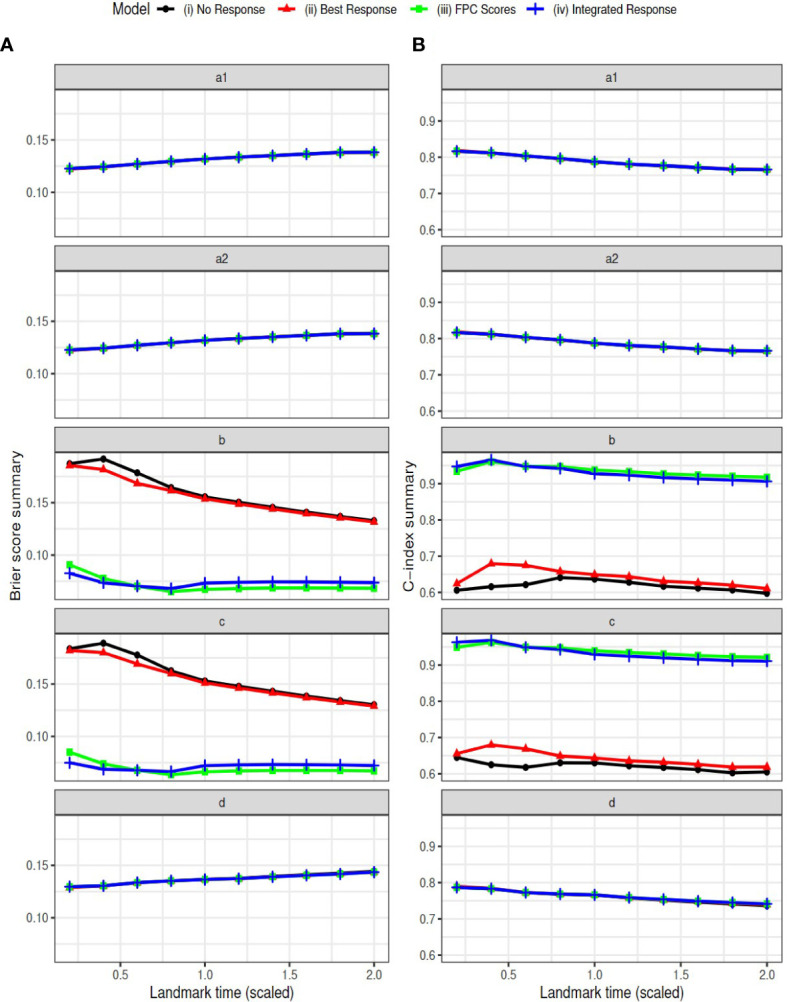
Simulation Results: Brier scores (column **A**) and C-index (AUC) (column **B**) in landmark analysis for the following simulation settings: direct effect only (settings a1 and a2), indirect effect only (setting b), both effects exist (setting c), and neither effect exists (setting d).

We selected the landmark time as a sequence of time from 0.2 to 2 with a step of 0.2 on the scaled time grids. Note that the summary variables for the longitudinal outcome were calculated at each landmark time based on available longitudinal data up to that time. The Brier scores and AUCs are reported in **Figure 4**. The estimated coefficients at landmark time points are summarized in the **Supplementary Materials**. The conclusions are similar to those based on the complete data. The models (iii) and (iv) have consistently better predictive ability (smaller Brier scores and larger AUCs) than model (i) and (ii) in settings ‘b’ and ‘c’. The four models have similar performance in the other three settings ‘a1’, ‘a2’ and ‘d’, where tumor response is assumed to have no effect on survival.

## 5 Discussion

Oncology studies routinely acquire measures of tumor burden longitudinally over the course of patient follow-up. This information is predominately dimension reduced to a binary tumor response variable indicating the occurrence of a partial or complete response as defined by the RECIST criteria. This article presented a landmark mediation survival model devised to estimate conjoint effects of treatment and longitudinal tumor assessments. Prediction performance was compared using different characterizations of tumor response following treatment. Conventional binary response based on the RECIST criteria was compared to analysis of the full longitudinal TB assessments using FPC scores as well as the integrated response. *R* ^2^ measures were adopted to quantifythe extent of treatment survival mediation effect attributable to longitudinal TB assessments. Implementation was demonstrated with two colorectal cancer studies: the Goldberg study comparing FOLFOX with IFL and the Amgen study on the additional effect of Panitumumab on FOLFIRI. The time-varying effects of treatment and baseline LDSUM were compared with models that leveraged different extent of information from the longitudinal tumor assessments. Prediction performance was compared using AUC. *R* ^2^ measures were adopted to quantifying the mediation effect of the longitudinal tumor burden.

We found that the longitudinal models with prediction based on FPC scores tended to yield larger AUCs when compared to models with conventional RECIST objective response. Moreover, it was discovered that the predictive utility of binary tumor response depends on the shape of the underlying longitudinal trajectories. With U-shaped trends for tumor burden following treatment, as observed in the Amgen study, binary objective response (PR or CR based on the RECIST criteria) was sufficient to characterize the most pertinent information contributed by the longitudinal data. AUCs obtained from models incorporating tumor response information were much larger than corresponding model absent tumor response.

In the absence of U-shaped trends in tumor burden over time, reducing the longitudinal TB data to a binary response discards important information regarding treatment survival mediation. Models using binary objective response applied to the Goldberg study, for example, yielded AUCs that were very close to those obtained in the model without tumor response. Based on the estimated *R* ^2^ and *SOS* effects, the longitudinal TB as defined by LDSUM presented no mediation effect in the Goldberg data. For the Amgen study, however, an estimated 35% to 70% of the treatment effect on OS was mediated through the pattern of longitudinal tumor assessments captured by FPC scores. Simulation demonstrated that FPCA based longitudinal predictors yielded smaller Brier scores and larger AUCs than the binary response model under all settings. Consequently, the complex relationships between treatment, survival, and tumor burden may be better elucidated with the widespread adoption of longitudinal analysis. FPC scores in particular offer a practical approach to synthesizing the longitudinal patterns with sufficient flexibility to capture the trends that describe treatment survival mediation.

Several limitations should be noted. The estimation approaches presented were founded on large sample theory which requires caution with the application to small sample data in practice. The interpretation of the mediation effect is uninterruptible when the estimated $R_{med}^{2}$ is negative or the resulted SOS effects are out of the [0,1] range. This may have resulted in our case study from the additional random variation induced by the joint model and/or the relatively small sample size of the Goldbergcase. Further investigation is required to define the minimal number of OS event one needs to observed before fitting mediation models for OS with longitudinal surrogates.

## Data availability statement

Publicly available datasets were analyzed in this study. This data can be found here: www.projectdatasphere.org.

## Author contributions

BH and PW contributed to conception and design of the study. JZ organized the database and performed the statistical analysis. JZ and BH wrote the first draft of the manuscript. All authors contributed to manuscript revision, read, and approved the submitted version.
