# Supplementary material for: Landmark mediation survival analysis using longitudinal surrogate
Source: Front Oncol. 2023 Jan 17;12:999324. doi: 10.3389/fonc.2022.999324 (PMC9887328; doi:10.3389/fonc.2022.999324)
Supplement: Supplementary file 1 [file DataSheet_1.pdf]

frontiers

Supplementary Material

1 ESTIMATED COEFFICIENTS IN SIMULATION STUDY

We present the estimated coefficients β_1 and β_2 in the simulation studies here for both the complete data and the landmark analysis. The mean and the first and third quartiles of the estimated coefficients β_1 and β_2 are plotted in Figire S1 for the complete data. We can see the mean estimates of the four models are close to the truth in settings 'a1', 'a2' and 'd', while models (iii) and (iv) have smaller bias than model (ii) in settings (b) and (c). The estimated coefficients in the landmark analysis are plotted in Figure S2. The solid lines are the mean of the estimates and the dashed lines are the first and third quartiles. In general we see the bias are small for all models in settings (a1), (a2) and (d), where no mediation effects exist. In setting (b) and (c), method (iv) has the smallest bias among all the models because it is the model we used to generate the data. Slight bias are observed in the estimated coefficients for other models in the landmark analysis. Generally we have FPC score based methods (iii) and (iv) have smaller bias than model (ii), which depends on binary response.

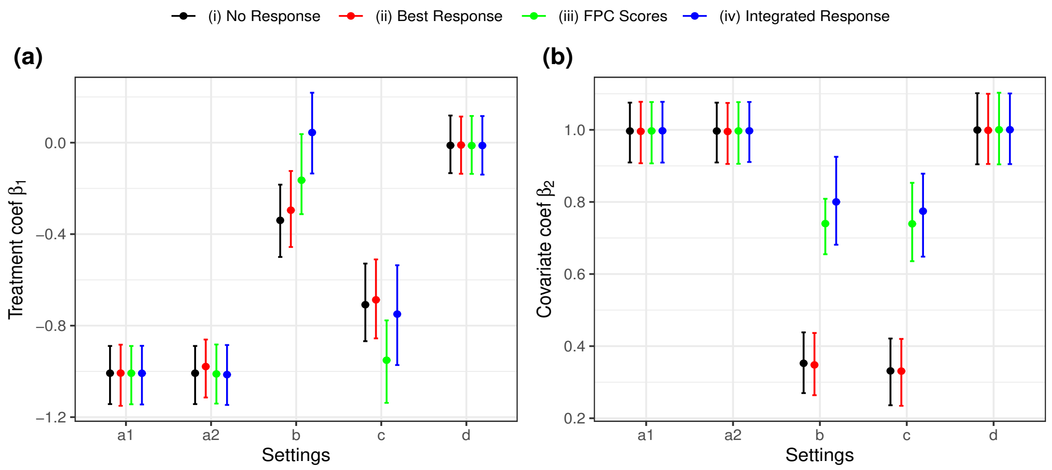

Figure S1. Simulation Results: coefficients estimation with complete dataset

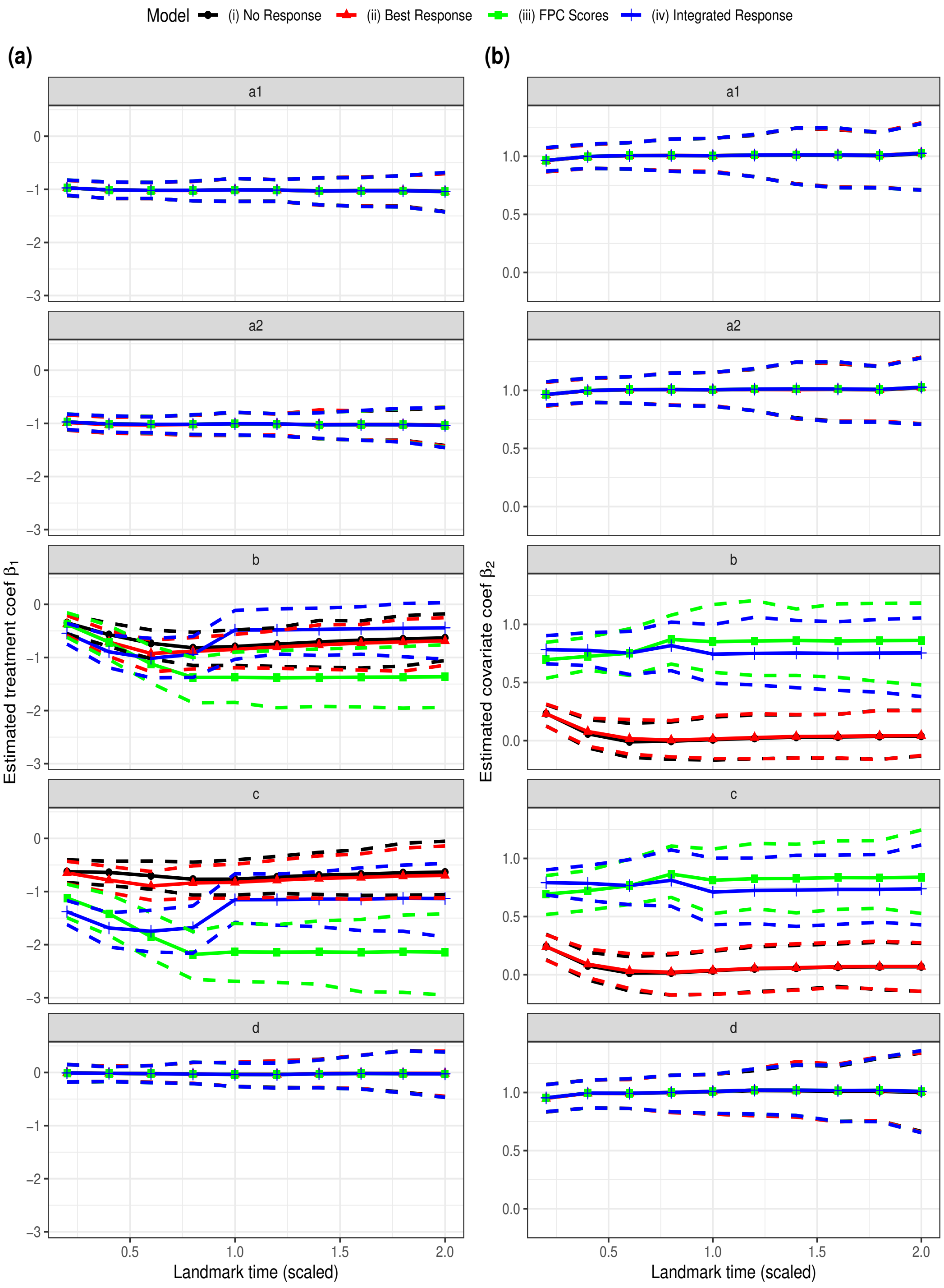

Figure S2. Simulation Results: estimated coefficients in landmark analysis (solid lines are the mean values and dashed lines are the 2.5% and 97.5% quantiles)
